# Neurological Manifestation of Recreational Fatal and Near-Fatal Diethylene Glycol Poisonings: Case Series and Review of Literature

**DOI:** 10.1097/MD.0000000000000062

**Published:** 2014-08-22

**Authors:** Yahia Zakaria Bashier Imam, Saadat Kamran, Hanfa Karim, Osama Elalamy, Tageldin Sokrab, Yasir Osman, Dirk Deleu

**Affiliations:** Neurology Section (YZI, SK, OE, TES, YO, DD); Department of Medicine (HK), Hamad Medical Corporation; and Weill Cornell Medical College in Qatar (SK, OE, TES, DD), Doha, Qatar.

## Abstract

Diethylene glycol is a common industrial solvent which is responsible for accidental and epidemic poisoning as early as the 1930s. Due to the unavailability and unaffordability of ethanol, people in Qatar among the low income group are consuming household chemicals, some of which contain diethylene glycol, for recreational purposes.

The history of ingestion is usually not volunteered and the initial clinical presentation is usually nonspecific, making it difficult to diagnose from the clinical presentation. Moreover, the biochemical profile varies with time, making the diagnosis more difficult. The neurological course and toxicity is less well characterized than its renal counterpart. Moreover, reports in the literature of such recreational poisoning is lacking particularly in the region.

Three cases of recreational diethylene glycol poisoning seen in Hamad General Hospital, Doha, Qatar from 2009 to 2012 are detailed here.

These illustrate the clinical course with emphasis on the neurological sequelae that include encephalopathy and multiple cranial and peripheral neuropathies with fatal and near-fatal outcomes. Neuroimaging in 2 were initially normal, but follow-up imaging showed brain atrophy. The third patient’s neuroimaging showed diffuse brain edema with evidence of transtentorial herniation. Nerve conduction studies were performed in 2 of the 3 cases and showed evidence of mixed sensorimotor neuropathy. The outcomes were death in 1 and severe neurological morbidity and disability in 2 cases.

Diethylene glycol is a dangerous substance when ingested and can result in mortality and severe morbidity, particularly from the renal and neurological manifestations. Whereas the mechanism of damage is less well known, the damage is likely dose related. The typical clinical pattern of evolution of the poisoning in the absence of cost-effective ways to detect it in the serum can help clinicians in making the diagnosis.

Neurological manifestations may include encephalopathy and multiple cranial and peripheral neuropathies with subsequent brain atrophy. Public awareness of the danger of such recreational use should be raised.

## INTRODUCTION

Diethylene glycol (DEG) is an alcohol formed by the condensation of 2 ethylene glycol molecules with an ether bond. It was first isolated in 1869 and put to commercial application as an industrial solvent in 1928.^1^ DEG is used as a solvent and an ingredient in numerous consumer products, including furnace fuel, antifreeze preparations, wallpaper strippers, and automotive chemicals. It is also found in several household products that do not have childproof packaging.

It has been linked to accidental, suicidal, and over a dozen epidemic poisonings, the earliest and most famous of which was the Massengill’s Elixir sulphanilamide incident in 1937 in America, which was the key for passing of the United States Federal Food, Drug, and Cosmetic Act in 1938.^2–5^ We report 3 cases of recreational DEG poisoning because of the consumption of furnace fuel with emphasis on the neurological findings. Literature review reveals paucity of reporting of such recreational use, particularly in the Middle East. We believe that this is the first report in the region.

## CASE REPORTS

### Case 1

A 40-year-old Sri Lankan man presented with a 1-day history of abdominal pain and vomiting. The initial vital signs and clinical examination were unremarkable except for low-grade fever of 38°C and bilateral loin tenderness. The patient denied any substance intake. Blood workup revealed renal failure, high anion gap metabolic acidosis, and leukocytosis (Table 1). The patient was treated with broad-spectrum antibiotics pending workup for sepsis, which turned out negative. The patient continued to have severe abdominal pain prompting computed tomography (CT) of the abdomen, which was also unremarkable. Five days later, the patient went into anuria with persistence of the high anion gap metabolic acidosis requiring dialysis. He developed labile hypertension and bilateral facial nerve palsy.

**TABLE 1:**
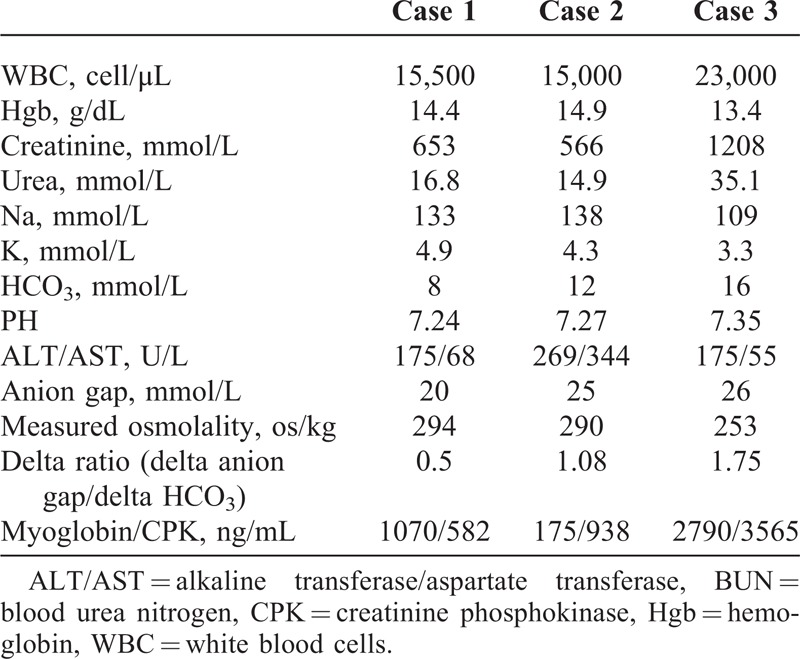
Showing the Chemical Profile of the 3 Cases

Cerebrospinal fluid (CSF) analysis performed at this time showed high protein of 0.98 g/L (normal 0.15–0.45 g/L), normal glucose, 45 cells/μL (normal 0–5 cells/μL) with 80% neutrophils, normal Gram stain, and cultures including tuberculosis and fungal cultures as well as a negative CSF viral panel. This was followed by bulbar symptoms and obtundation requiring intubation and assisted ventilation. CT head at that time was normal. An electroencephalogram (EEG) revealed moderate to severe slowing in the theta and delta ranges, but no epileptiform discharges.

By that time, the patient’s friend volunteered information about the patient drinking 150 mL of a clear colorless liquid for recreational purposes. The empty bottle they brought was of furnace fuel composed of 100% DEG solution.

**FIGURE 1:**
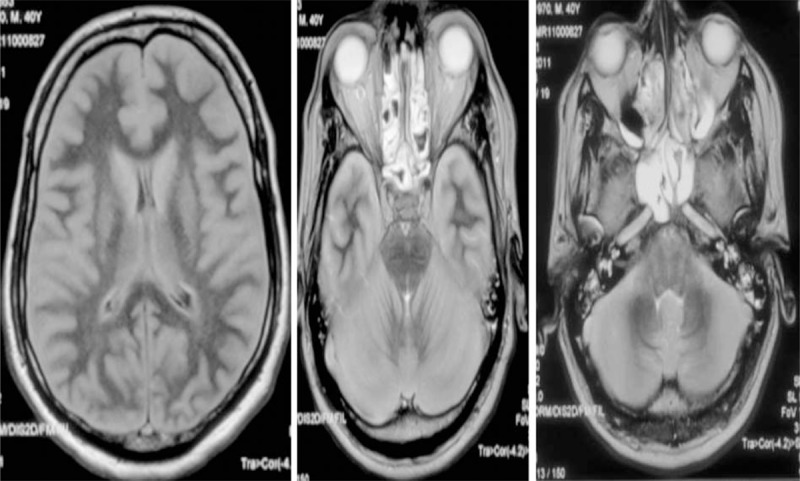
FLAIR MRI showing cerebral edema with thickening of the gray matter. Loss of ambient cistern and effaced 4th ventricle. FLAIR = fluid attenuated inversion recovery.

The patient went into deep coma, aspiration pneumonia, and acute respiratory distress syndrome within 5 days. Magnetic resonance imaging (MRI) of the brain showed diffuse brain edema and transtentorial herniation (Figure 1). Twenty hours later the patient went into cardiac arrest and died.

### Case 2

A 21-year-old Nepalese man presented to the emergency department with a 2-day history of abdominal pain and repeated vomiting. The initial vital signs and the clinical examination were unremarkable except for diffuse vague tenderness in the abdomen. Laboratory studies (summarized in Table 1) revealed renal failure and high anion gap metabolic acidosis. An urgent CT abdomen revealed mild perirenal fat stranding. Urine microscopy was unremarkable. On day 5 of the illness, clinical examination revealed bilateral facial nerve palsy. Subsequently, the patient developed bulbar symptoms as well as bilateral palsies of cranial nerves III, IV, and VI. He had distal neuropathic pains associated with distal lower limb weakness, diminished reflexes, and downgoing plantars. Brain CT scan was normal. Upon repeated questioning, the patient gave a history of ingestion of around 60 mL of furnace fuel. The high anion gap metabolic acidosis persisted and the patient went in anuria requiring hemodialysis (HD). Brain MRI done at this stage (nearly 2 weeks after ingestion) was essentially normal including magnetic resonance spectroscopy (MRS). Ten days after admission, a nerve conduction study (NCS) was performed, which showed predominately moderate axonal sensorimotor peripheral neuropathy (Table 2). Subsequently, he developed aspiration pneumonia and was transferred to the medical intensive care unit (MICU) with a decrease in the level of consciousness. He was intubated and treated with intravenous antibiotics. An EEG revealed moderately severe slowing in the theta and delta ranges, but no epileptiform discharges. Eventually, the patient underwent tracheostomy and was weaned off the ventilator but remained HD dependent. He was fully conscious and cooperative but had residual facial paresis, dysphonia, and moderate weakness of all 4 limbs with bilateral foot drops as well as persistent areflexia.

**TABLE 2:**
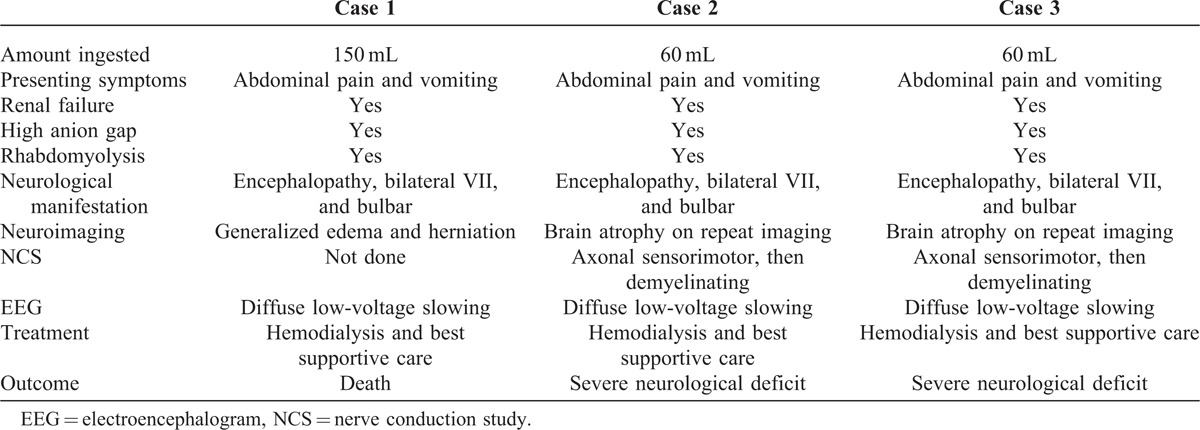
A Comparison Between the 3 Cases

**FIGURE 2:**
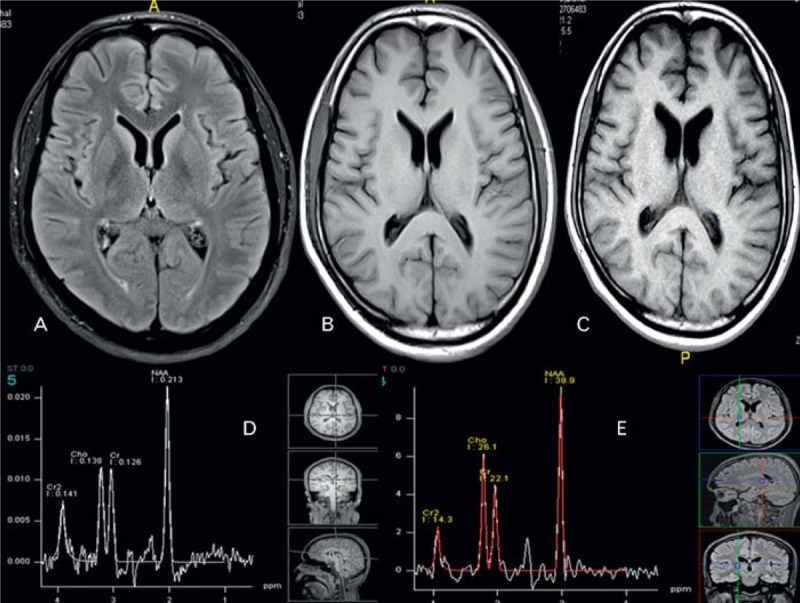
Repeat brain MRI after 3 months. (A) FLAIR MRI shows no abnormal signal (B) and (C), T1-WI during and after coma showing mild atrophy (see Table 3). MRS (D) and (E) 3 months apart (during coma and after) normal. FLAIR = fluid attenuated inversion recovery, MRS = magnetic resonance spectroscopy, T1-WI = T1 weighted image.

Repeat brain MRI (Figure 2) after 3 months showed evidence of mild generalized brain atrophy (Table 3). Repeat NCS showed bilaterally absent conduction in tibial, peroneal, and sural nerves. The upper limbs showed a predominately demyelinating sensorimotor neuropathy. Visual evoked potentials (VEPs) and brain stem auditory evoked potentials (BAEPs) were normal.

**TABLE 3:**
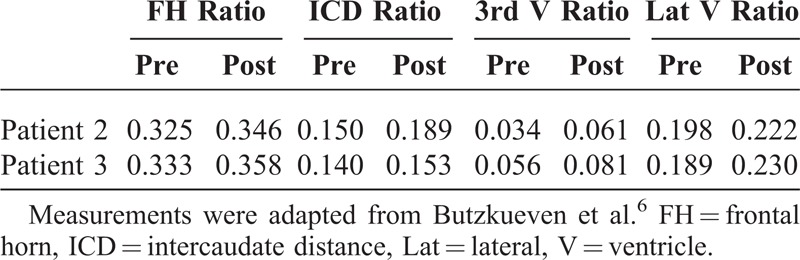
Demonstrating Objective Atrophy Pre and Post Coma

### Case 3

A 21-year-old Nepalese man presented 2 days after case number 2 with similar complaints of abdominal pain and vomiting. Clinical examination was only remarkable for diffuse vague tenderness in the abdomen. Laboratory studies (summarized in Table 1) revealed renal failure and high anion gap metabolic acidosis. The patient was admitted to the ward as a case of acute kidney injury and possible rhabdomyolysis. Slow correction of hyponatremia was carried out, his urine output and renal parameters were worsening, and the high anion gap metabolic acidosis persisted. HD was started 2 days later.

Four days after admission, the history of ingestion of around 60 mL of furnace fuel (same as in Case 2) was revealed.

Subsequently, he developed bilateral cranial nerve palsies including VII, VIII, IX, and X cranial nerves. Further neurological examination revealed quadriparesis, global areflexia, and equivocal plantars. Brain CT was normal. The patient developed pulmonary edema warranting urgent intubation, mechanical ventilation, and HD. He was transferred to the MICU for further care. An EEG revealed moderate to severe slowing of the background pattern without epileptiform activity.

Follow-up at 3 months showed that the patient remained dependent on HD.

He had persistent bilateral facial paresis, bulbar palsy, and foot drops, but otherwise intact cognitive functions.

**FIGURE 3:**
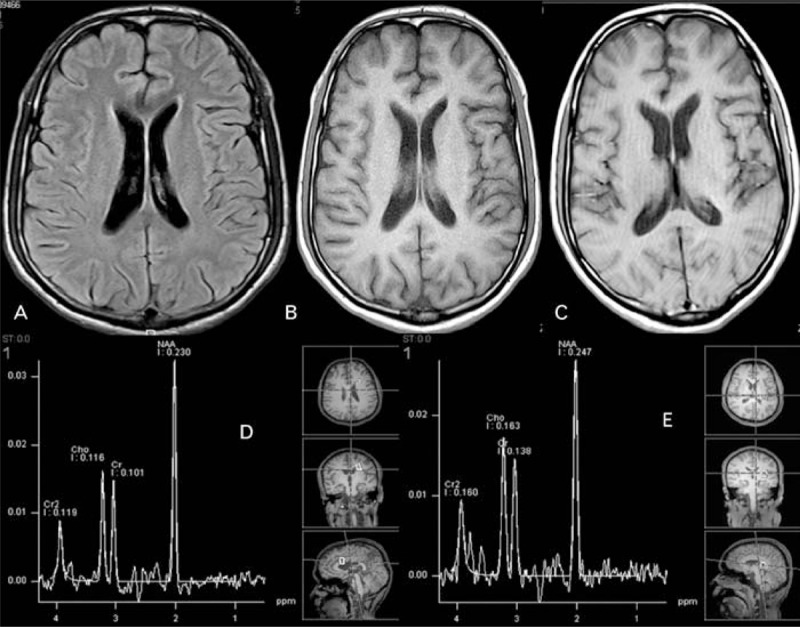
Follow-up brain MRI. (A) FLAIR MRI shows no abnormal signal (B) and (C), T1-WI during and after coma showing mild atrophy (see Table 3). MRS (D) and (E) 3 months apart (during coma and after) normal. FLAIR = fluid attenuated inversion recovery, MRS = magnetic resonance spectroscopy, T1-WI = T1 weighted image.

Follow-up brain MRI (Figure 3) showed mild generalized cerebral atrophy (Table 3) and a normal MRS. VEPs and BAEPs were normal. Follow-up NCS showed worsened mixed sensorimotor demyelinating neuropathy, with absent conduction in the lower limbs.

## DISCUSSION

Alcohol has been used as a recreational substance as early as the stone age.^7^ Through alcohol dependence, liver cirrhosis, cancers, and injuries occur (either directly or through dangerous actions such as drunk-driving, violence as well as the impact of maternal drinking on fetal and child development); alcohol possess a big global socioeconomic burden.^8^

Influenced by cultural and religious factors such prohibition of alcohol consumption in Islam, alcohol is difficult to obtain in the Middle East in a cheap manner. Hence, individuals in low social ranks tend to use alcohol substitutes instead of ethanol for recreational purposes.

DEG is a common industrial solvent that is responsible for accidental and epidemic poisoning as early as the 1930s. A few reports of recreational use are reported in the literature with various outcomes.^3,4^ Its sweet taste and omnipresence may be contributing factors to its use as an ethanol substitute.

Of particular concern are the renal and neurological toxicities that resulted in high morbidity and mortality.^1,3,4^

DEG is metabolized into 2 primary metabolites, 2-hydroxyethoxyacetic acid (2-HEAA) and diglycolic acid (DGA).^9^ Much of its toxicity is attributed to 2-HEAA.^1,3^ Recently, Landry et al^9^ suggested that the intracellular uptake of the other metabolite DGA is the cause of toxicity, particularly in the kidneys. A recent report^10^ on human samples from the Panama DEG mass poisoning has further implicated these 2 metabolites with the detection of 2-HEAA and DGA in the sera and DGA in the urine of cases. This was consistent with prior animal data.^11^

Interestingly, DGA and HEAA were significantly present in CSF samples of cases and not controls, which may be linked to the symptoms and signs of neurotoxicity. The exact mechanism of neurotoxicity, however, remains unknown.

### Clinical, Neurophysiological, and Histopathological Features

The clinical course follows a predictable triphasic pattern: the first phase is the gastrointestinal phase, notably abdominal pain, nausea, and vomiting, followed by phase 2 which is characterized by renal involvement in the form of decreased urine output and high anion gap metabolic acidosis, which may progress to anuria and the need for HD.

This is followed by a delayed progressive neurological syndrome that is characterized by encephalopathy and multiple cranial and peripheral neuropathies that occur 5 to 20 days after the ingestion, if the patient survives the initial phases.^12^ These phases tend to overlap and the severity of symptoms is correlated to the amount ingested.^1^

This is similar to the delayed neurological sequelae seen with stage IV massive ethylene glycol poisoning.^13^ However, the difference in metabolism and the absence of oxalate crystalluria or oxalate deposition in sections of tissue examined^14,15^ favor that toxicity may be through different mechanisms.

The development of severe renal failure requiring HD seems to predict the progression into the neurological phase.

The neurological manifestation was first reported by Wordley^16^ in 1947 when he described 2 cases. One patient drank half a bottle of pure DEG and went into deep coma. His necropsy showed macroscopically congested brain, regrettably however, it was never examined microscopically. The other case was a patient who had only a few mouthfuls and developed headaches, vomiting, repeated seizures, and renal failure. He was managed successfully with decapsulation of his kidneys.

Other reports in the literature are summarized in Table 4.

**TABLE 4:**
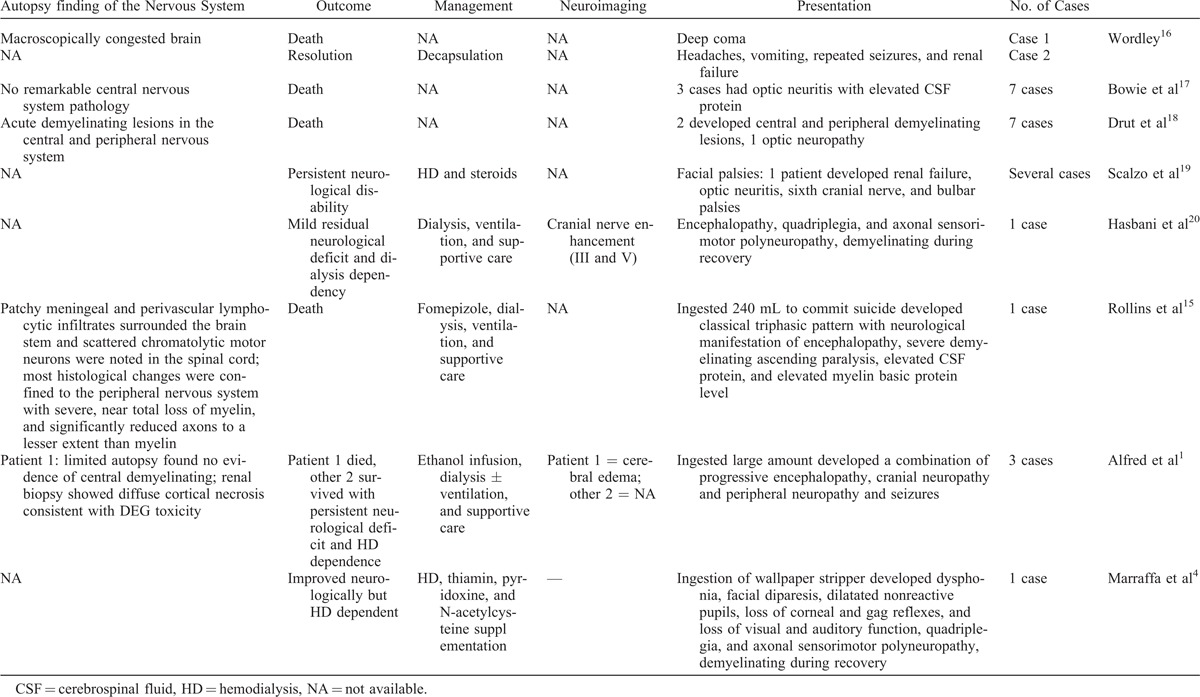
Summarizing Cases With Neurological Findings in the Literature

One notices that neurological signs in the 2 surviving initial presenters (Alfred et al^1^) improved over 4 to 6 months, but they remained dialysis dependent, similar to our patients 2 and 3. This suggests that the severity of renal insult likely predicts the development of neurological sequelae.

Additionally, a report from China in 2009 by Luo et al^21^ gave us some insight into what happens upon direct intravenous toxicity as opposed to oral ingestion. They described the effects of DEG poisoning on patients with liver disease receiving intravenous armillarisin-A. The report provided details of close follow-up, dose administered, and DEG levels. Out of 64 patients, 15 patients exhibited features of toxicity, mostly following the classical triphasic pattern. Of these 15 patients, 10 developed neurological manifestations after a mean of 14 days, including the involvement of second, third, seventh, and ninth cranial nerves, peripheral neuropathy, encephalopathy, limb paralysis, and respiratory muscle paralysis in severe cases. Nine out of these 10 patients died. The authors have suggested the reduction in the activity of alcohol dehydrogenase (ADH) and acetaldehyde dehydrogenase enzymes in patients with liver disease as a possible mechanism leading to poisoning. Additionally, and counterintuitively, though the patients had liver disease, their liver function remained unchanged after the poisoning, which “suggests that DEG has no impairment on the hepatobiliary system and administration of DEG by venous route may not be metabolized in the liver.”^21^

The largest series on neurological toxicity by far was reported recently by Sosa et al,^22^ detailing findings from 40 out of 46 cases (87%) from the Panama mass poisoning of 2006. The most common neurological finding was limb weakness in 31 (78%) of whom almost all had reduced or absent deep tendon reflexes. More than half of the patients had lower motor neuron facial palsy. Ten patients (22%) developed progressive flaccid paralysis culminating in quadriparesis. Sixty three percent developed signs suggestive of autonomic dysfunction. Nerve conduction on 21 patients with limb weakness showed evidence of severe sensorimotor axonal neuropathy in 19. Demyelination of spinal roots and cauda equina was seen on autopsies of 5 neurologically affected cases.

Our patients followed the classical triphasic pattern of the disease, and it was this pattern of evolution that led us to further inquire and uncover the nature of ingestion. Furthermore, encephalopathy, facial diplegia, and bulbar symptoms dominated the picture earlier on with peripheral neuropathy setting in and evolving into axonal injury, then subsequently transforming into a demyelinating pattern upon improvement later on, similar to our patients 2 and 3.

### Neuroimaging

The advancement in neuroimaging techniques made possible the diagnosis of many toxic brain injuries during life, as opposed to autopsy. However, DEG toxicity appears to differ from other intoxications that have distinct patterns on MRI.

Methanol intoxication, for example, targets the putamina and causes necrosis and variable degrees of hemorrhage,^23^ whereas ethylene glycol intoxication causes symmetric involvement of the basal ganglia, thalami, amygdala, hippocampus, brain stem, and white matter tracts.^23^ This does not seem to be the case in DEG poisoning. Reports in the literature are scarce at best.

Brain CT scans failed to show any abnormality in our cases, whereas the brain MRI was initially normal in the 2 surviving cases and showed evidence of brain edema with herniation in the patient who died. Generalized brain edema may be a surrogate marker for severe intoxication and seems to be an ominous sign if present. Alfred et al^1^ described this finding in a patient with severe intoxication who died. However, others have reported fatalities with a normal brain MRI.^15^ Follow-up brain MRI in our surviving cases showed mild degrees of atrophy similar to what Scalzo^19^ reported. The clinical significance of this finding remains to be elucidated. MRS failed to show any abnormality in those 2 cases, which raises the question of how the encephalopathic process and the ensuing atrophic changes came about pathophysiologically.

Contrast enhancement of several cranial nerves was reported by Hasbani et al^20^ and is in keeping with the clinical picture of multiple cranial neuropathies. Administration of contrast was not justified in our cases given the presence of severe renal failure.

### Treatment

Treatment is aimed at the early phases of poisoning in the form of decontamination, giving an antidote such as fomepizole, to reduce conversion of DEG to its toxic metabolites and HD or a combination of the above.^24^ Fomepizole acts primarily as an ADH inhibitor. Because DEG is thought to be initially metabolized by ADH, the metabolites of DEG, but not DEG itself, are responsible for the observed toxicity.^10,11^

Blocking the metabolism of DEG via concomitant administration of the ADH inhibitor fomepizole should theoretically prevent target organ toxicity.^11^ DEG being water soluble, has low molecular weight and is dialyzable; thus, HD still plays a major role in DEG toxicity management.^24^ In these 3 cases that we have described, dialysis was initiated rather late at the stage of anuria. This is likely because the history of ingestion was not volunteered and the awareness of DEG poisoning is low among the treating physicians.

Given the mortality and morbidity of such poisoning coupled with the short half-life of about 3 hours, a high index of suspicion and rapid institution of treatment is required.^24^ The diagnosis should be considered in patients presenting with nausea, vomiting, abdominal pain, gap acidosis, and evolving acute renal failure.

Unfortunately, once the delayed neurological stage is reached only supportive treatment for DEG toxicity remains.^3,24^

### Outcome

Outcomes after reaching the delayed neurological phase varies from fatal to severe disability and rare clinical resolution with minor residual deficits. Dialysis dependence seems to be the rule however. Outcomes are influenced by premorbid conditions of the patients, the amount ingested, and the promptness of the recognition and institution of treatment of the poisoning.^1,3,15^

Case fatality rates are variable and range from 59% to 98% depending on the amount of ingestion, severity of clinical features, and access to tertiary hospitals, intensive care, and renal replacement therapy.^22^ Death was reported due to severe cardiovascular complications, including arrhythmias and profound hypotension consistent with autonomic dysfunction.

Long-term follow-up of survivors of DEG positioning for up to 18 months was recently published.^25^ It reported a mortality of 15.6% at 18 months and a tendency toward neurological improvement. Renal improvement was also observed in the nondialysis-dependent patients. However, no delayed neurological or renal sequelae was noted.

## CONCLUSION

DEG is a dangerous substance if ingested and can result in mortality and severe morbidity, particularly from the renal and neurological manifestations. Although the mechanism of damage is less well known, the damage is likely dose related. The typical clinical pattern of evolution of the poisoning, in the absence of cost-effective ways to detect it in the serum, can help clinicians in making the diagnosis.

Neurological manifestations may include encephalopathy and multiple cranial and peripheral neuropathies with subsequent brain atrophy. Public awareness of the dangers of such recreational use should be raised.
